# Focus On: Biomarkers of Fetal Alcohol Exposure and Fetal Alcohol Effects

**Published:** 2011

**Authors:** Ludmila N. Bakhireva, Daniel D. Savage

**Keywords:** Maternal alcohol exposure, prenatal alcohol exposure, pregnancy, fetal alcohol effects, fetal alcohol spectrum disorders, prenatal diagnosis, screening and diagnostic method, biomarkers

## Abstract

One of the ongoing challenges for the accurate diagnosis and treatment of children with fetal alcohol spectrum disorders (FASD) is the difficulty of confirming whether a mother drank during her pregnancy. Commonly used screening questionnaires often are unreliable, and current established biomarkers of alcohol consumption are not sensitive enough for use with many pregnant women. These limitations underscore the critical need to develop novel biomarkers with greater sensitivity for detecting moderate levels of drinking during pregnancy for longer periods of time after the last drinking episode. In addition, developing reliable biomarkers of fetal alcohol effects that can identify children at risk for adverse neurobehavioral outcomes could lead to behavioral interventions earlier in development. The use of animal models of FASD in biomarker development could accelerate progress in this challenging field of research.

Despite public-awareness campaigns and warning labels on alcoholic beverages, roughly one of every eight women in the United States continues to drink during her pregnancy. The term fetal alcohol syndrome (FAS) was first used in the 1970s to describe a specific pattern of malformations associated with prenatal alcohol exposure (Jones et al. 1974). The continuum of clinical presentations caused by prenatal alcohol exposure is known as fetal alcohol spectrum disorders (FASD). Attempts to accurately estimate the prevalence of FASD have been challenging because of numerous methodological issues, including diagnostic criteria, passive versus active surveillance, and difficulties with ascertainment of milder cases of FASD in the absence of fetal alcohol–induced birth defects. The prevalence of FAS ranges from 0.5 to 7.0 per 1,000 live births in the general population and up to 9.8 per 1,000 live births in high-risk groups (May et al. 2001, 2009). The prevalence of the entire continuum of FASD might be as high as 1 to 5 percent in young school children in the United States (Lupton et al. 2004; May et al. 2001, 2009), which is higher than the prevalence of autism spectrum disorders.

A large majority of fetal alcohol–affected children may show no physical evidence of prenatal alcohol–associated birth defects. In the absence of characteristic FAS dysmorphology, recognition of FASD requires confirmation of maternal drinking during pregnancy. However, maternal drinking histories are generally unreliable, and the utility of currently available biomarkers of alcohol exposure is limited. Thus, one of the critical challenges for the fetal alcohol research community is to develop better methods for detecting drinking during pregnancy. This article will examine current methods for detecting maternal and fetal alcohol exposure, including self-reporting and biomarkers. The limitations of current methods, however, underscore the critical need to develop new biomarkers with greater sensitivity for detecting moderate levels of drinking during pregnancy for longer periods of time after the last drinking episode. Thus future research needs are reviewed as well.

The development of more sensitive and reliable biomarkers for alcohol use could serve two important objectives in clinical intervention. First, the ability to identify more women who are drinking during pregnancy, particularly early in pregnancy, would allow providers to educate such patients about the dangers of drinking during pregnancy and provide counseling to reduce the risk of subsequent drinking episodes. Effective intervention at this point would benefit not only the mother and fetus but also could reduce the risks of fetal alcohol damage in subsequent pregnancies. Second, a biomarker, or perhaps a combination of biomarkers and other clinical measures, could provide an early indication of whether a newborn child is at risk for developing behavioral and/or cognitive problems later in life, creating opportunities for earlier postnatal interventions that may reduce longer-term adverse consequences. Early intervention programs, such as targeted sociocognitive programs focused on improving behavior and math functioning (Kable et al. 2007), language and literacy training (Adnams et al. 2007), and rehearsal training to address working-memory deficits (Loomes et al. 2008), have been shown to reduce the social and behavioral difficulties experienced by fetal alcohol–affected children (see Paley and O’Connor, pp. 64–75, in this issue). However, in the absence of reliable prognostic indicators, the adverse effects of fetal alcohol exposure may go unrecognized for years, diminishing the benefits afforded by earlier intervention.

An ideal biomarker for detecting alcohol use among pregnant women should have the following attributes: (1) the capacity to detect low-to-moderate levels of drinking over extended periods of time after the last drinking episode; (2) a high probability of accurately detecting drinking that occurred during pregnancy (i.e., high sensitivity); and (3) a low rate of false-positive test results (i.e., high specificity). In addition, important technical considerations include (1) a biological sample easily obtained by a minimally invasive and clinically acceptable procedure that (2) requires little or no sample preparation, and (3) a simple, relatively inexpensive analytical procedure that (4) provides rapid results, ideally in a point-of-care setting. Unfortunately, none of the current alcohol biomarkers adequately satisfies more than one or two of these attributes.

## Current Approaches for the Detection of Fetal Alcohol Exposure

### Self-Reporting Methods

#### Screening Questionnaires

Despite growing interest in biomarkers that can objectively and accurately identify alcohol exposure in mothers and children, maternal self-report remains, by far, the most prevalent approach used in clinical practice for identifying people with hazardous or harmful alcohol consumption. Screening questionnaires such as TWEAK,^1^ the Alcohol Use Disorders Identification Test (AUDIT), the Michigan Alcohol Screening Test, and CAGE^2^ are designed to assess a history of alcohol-related behaviors (Russell et al.1996). Such questionnaires are brief and easy to administer in clinical settings and might be quite sensitive for identifying risky drinkers. Given the existing body of literature, TWEAK and AUDIT appear to be the most sensitive screening questionnaires for identifying risky drinking (14 or more drinks per week) in pregnant women, with sensitivities greater than 90 percent and 70 percent, respectively (Chang et al. 1999; Russell et al. 1996). However, such questionnaires do not capture the amount, pattern, or timing of alcohol consumption.

#### The Timeline Follow-Back Procedure

A more extensive interview technique, known as the timeline follow-back (TLFB) procedure (Sobell and Sobell 1992) currently is considered a gold standard for assessing alcohol exposure. During the TLFB interview, patients are asked to report exact quantities of the specific alcoholic beverages consumed each day during the defined timeframe. To minimize recall bias, questions are linked to specific days, events, and activities during the reported period. Sensitivity and specificity of novel biomarkers are traditionally established relative to quantities of alcohol consumption (absolute ounces of alcohol per day and per drinking day) reported on the TLFB interview. A more objective gold standard might be the actual diagnosis of FAS, partial FAS, or alcohol-related neurodevelopmental disorder (ARND) in affected children; however, such a gold standard would have its own methodological limitations, given that most children are not diagnosed with FASD until they start exhibiting cognitive and behavioral problems in school. Furthermore, general pediatricians often lack expertise to recognize FASD (Jones et al. 2006). In addition, not all children prenatally exposed to alcohol develop FASD.

## Laboratory Tests

Given the inherent difficulties of self-report, including inaccurate recall, embarrassment, and fear of stigmatization, biomarkers of chronic and recent alcohol exposure taken individually and in combination may provide better corroboration of maternal drinking history.

### Biomarkers of Alcohol-Induced Pathology

The most established serum biomarkers of alcohol-induced pathology are γ-glutamyltransferase (GGT), mean corpuscular volume (MCV), and carbohydrate-deficient transferrin (CDT). GGT is a liver enzyme elevated in blood as a result of chronic exposure to alcohol. MCV is a measure of average red blood cell volume, which increases with chronic alcohol consumption over a 1-to 3-month period. GGT is reported to be a better marker of heavy alcohol consumption in pregnant women because MCV is physiologically elevated during the second half of pregnancy (Barrison et al. 1982; Halmesmaki et al. 1992; Ylikorkala et al. 1987). However, although MCV has limited sensitivity during the second and third trimesters of pregnancy, it is a more specific biomarker than GGT (Sarkola et al. 2000; Ylikorkala et al. 1987). The half-life for detecting elevated GGT is 2 to 3 weeks compared with 17 weeks for MCV, making GGT a better marker to detect recent changes in drinking patterns.

CDT is a modified form of the iron-transporting protein transferrin and is a more specific measure of alcohol consumption than GGT or MCV. CDT is a reliable marker of current heavy drinking that is usually elevated after an intake of 50 to 80 grams of alcohol (4 to 6 standard drinks) per day during the previous 1 to 3 weeks (Allen et al. 1994; Helander and Jones 2000). However, because CDT has a relatively short half-life, its levels usually normalize after 4 weeks of abstinence (Allen et al. 1994). In pregnant women, total transferrin increases with advanced gestational age; therefore, CDT currently is measured as a percentage of total transferrin (percentage CDT).

### Biomarkers of Alcohol Metabolism

The most sensitive and specific measures for detecting drinking are the presence of alcohol in body fluids or exhaled breath or the presence of alcohol’s most common metabolite, acetaldehyde, in blood. However, the short half-lives of alcohol and acetaldehyde limit their utility in clinical practice, especially among pregnant women who may have more intermittent episodes of alcohol consumption. The following sections review the potential use of other alcohol metabolites as biomarkers.

#### Fatty Acid Ethyl Esters

Fatty acid ethyl esters (FAEEs) are nonoxidative metabolites that are formed when alcohol conjugates to endogenous free fatty acids and fatty acyl-CoA (Laposata 1998). The sensitivity of FAEEs extracted from meconium, the first stool of a newborn, for the identification of prenatal alcohol exposure varies from 26.9 percent to 100 percent across studies (Bearer et al. 1999, 2003; Chan et al. 2003; Derauf et al. 2003; Moore et al. 2003; Ostrea et al. 2006). These variations in sensitivity may be attributed to difficulties in the extraction of FAEEs from complex media, differences in the study populations, and potential confounders, such as diet. However, the advantages of meconium FAEE analysis include the ability to identify moderate drinking (Bearer et al. 1999) and a greater sensitivity for identifying binge-drinking patterns rather than average consumption per week (Bearer et al. 2003). In addition, the presence of FAEEs in meconium has been reported to be predictive of subsequent mental and psychomotor delays in children (Peterson et al. 2008). Despite these advantages, the collection of meconium can be challenging in a clinical setting for the following reasons:(1) the limited window for specimen collection (usually the first 48 hours after birth); (2) the unavailability of the sample in up to 10 percent of newborns, especially preterm infants; and (3) the need to freeze the specimen at ultralow temperatures within the first 12 hours (later freezing is acceptable, but it can diminish FAEE levels). Finally, the validity of meconium FAEEs in identifying risky and binge drinkers who discontinued or substantially decreased alcohol use later in pregnancy is unknown.

#### Ethyl Glucuronide and Ethyl Sulfate

Ethyl glucuronide (EtG) is a metabolite of alcohol produced through a reaction with glucuronic acid in the liver. EtG can be detected in urine for up to 5 days after a drinking episode (Wurst et al. 2005). Studies evaluating the validity of EtG for the identification of alcohol abuse and/or social drinking among pregnant women are lacking. Results of a pilot study (Wurst et al. 2008*b*) in Sweden indicate that screening for EtG and FAEEs can identify more potential alcohol consumers among pregnant women than the AUDIT questionnaire alone, but the sensitivity of this test in pregnant women has not been established. Among nonpregnant patients, EtG reached a sensitivity of 83.5 percent for the identification of recent (within 4 days) alcohol consumption (Wurst et al. 2004*b*). One drawback of EtG’s high sensitivity is the potential for false-positive results, which can be produced as a result of exposure to alcohol-containing hand sanitizers, mouthwashes, cough syrups, and some beauty products (Kissack et al. 2008). In addition, false-negative results also can result from the hydrolysis of EtG by *E. coli* in patients with urinary tract infections, which are particularly common among pregnant women.

Ethyl sulfate (EtS) is another alcohol metabolite produced by a reaction in the liver (Helander and Beck 2005). Although it is not sensitive to bacterial hydrolysis (Helander and Dahl 2005), it has a shorter detection period in urine (up to 30 hours after alcohol ingestion) compared with EtG (Kissack et al. 2008). Both EtG and EtS concentrations can be significantly affected by water-induced diuresis; thus, they need to be expressed relative to the creatinine value to compensate for urine dilution (Helander and Beck 2005). Given the limitations of each test, a combined EtG/EtS battery can improve the validity of each individual biomarker for identification of recent drinking. The validity of these tests in pregnant women is largely unknown, however. In a pilot study conducted among pregnant women in Sweden, only one woman tested positive for urine EtG, and none were positive for EtS among nine women who stated that they consumed alcohol during their ongoing pregnancy (Wurst et al. 2008*b*). Thus, despite the high sensitivity of EtG and EtS, their major limitation is the short detection period.

#### Phosphatidylethanol

Phosphatidylethanol (PEth) is a phospholipid formed in the presence of alcohol that is detectable in blood up to 3 weeks after sustained alcohol intake (Hannuksela et al. 2007). Among nonpregnant patients with alcohol dependence, PEth demonstrated a 94.5 percent (Hartmann et al. 2007) to 99 percent (Aradottir et al. 2006) sensitivity. It also appears to be sensitive for the identification of moderate drinking (less than 40 grams per day) in nonpregnant women (Aradottir et al. 2006). A recent study (Steward et al. 2010) among women of reproductive age demonstrated that PEth was detectable in 93 percent of women averaging more than two drinks per day but in only 53 percent of women consuming up to one drink per day. In that study, the correlation between PEth and total alcohol consumption in the past 2 weeks was modest but statistically significant. PEth and EtG combined were able to identify alcohol-consuming patients who scored below the cutoff on AUDIT in an emergency-room setting (Kip et al. 2008). However, the validity of PEth has yet to be established, particularly in pregnant women who consume moderate amounts of alcohol.

## Recent Developments in Biomarkers for Fetal Alcohol Exposure

With the exception of measuring FAEEs in meconium, most work on alcohol biomarkers to date has focused on the use of maternal blood, serum, or urine samples as the biological sample source. More recently, investigators have begun examining the utility of other tissue types as sources for alcohol biomarker detection. These include hair, fingernails, umbilical cord tissue, placenta, and newborn blood. There is some evidence to suggest that these tissue types may serve as repositories for biomarkers of alcohol metabolism, providing detectable measures of alcohol consumption over longer periods of time. Another advantage of these tissue types is their relative ease and safety in sample collection. However, sample preparation and the extraction of alcohol biomarkers from these tissues generally is more challenging, compared with preparation and extraction from samples described above. In addition, to date, very limited data are available regarding the validity of biomarker analyses in these tissue types for identifying drinking during pregnancy.

### Analysis of Biomarkers in Maternal and Newborn Hair

Hair FAEE analysis conducted among nonpregnant alcoholic patients in a detoxification program or among heavy drinkers demonstrated 90 to 100 percent sensitivity and 90 percent specificity (Pragst and Yegles 2008; Wurst et al. 2004*a*). However, very low FAEE concentrations were detected even among strict abstainers, possibly as a result of the use of hair cosmetics or endogenous alcohol resulting in false-positive results (Pragst and Yegles 2008). In a guinea pig model of fetal alcohol exposure, alcohol-exposed offspring demonstrated a 15-fold-higher cumulative FAEE level in hair compared with control animals (Caprara et al. 2005*a*). However, researchers conducting a clinical study (Caprara et al. 2005*b*) reported that mild infrequent maternal drinking does not elevate FAEEs in neonatal scalp hair. Thus, the pattern of alcohol consumption that can be captured by FAEE analysis in neonatal hair has yet to be determined.

In a study of nonpregnant methadone-maintenance patients, Wurst and colleagues (2008*a*) reported that EtG demonstrated a relatively high correlation with AUDIT scores and self-reported alcohol intake in the previous 7 days. However, the sensitivity and specificity was not reported. In a pilot study among pregnant women in Sweden, only 12.5 percent of those positive for hair EtG admitted ongoing drinking on the AUDIT questionnaire (Wurst et al. 2008*b*). This raises questions of either severe underreporting on screening questionnaires or a high false-positive rate with hair EtG testing.

Although hair analysis provides important advantages over other biological tissues, it also has certain limitations. As noted above, there is no clear correlation between the dose of alcohol consumed and concentrations of FAEEs or EtG in hair (Pragst and Yegles 2008). In addition, differences in hair growth and hair care can affect results. Furthermore, the technically challenging microextraction of biomarkers from hair is not routinely performed by clinical laboratories and is expensive, limiting widespread clinical use. Moreover, collection of hair, especially neonate hair, can be problematic because of aesthetic reasons and cultural practices.

### Analysis of Biomarkers in Newborn Blood

Dried blood spot (DBS) analysis, collected from a neonatal heel stick, was first introduced in 1963 to test for the metabolic genetic disorder, phenylketonuria, in newborns (Guthrie and Susi 1963). Since then, Guthrie card samples—DBS on a filter paper—are routinely collected from more than 95 percent of newborns Nationwide and are used to detect congenital conditions, infectious disease, and DNA/RNA analyses (Clinical and Laboratory Standards Institute 2007). DBS collected for routine newborn screening have been used for illicit-drug testing (Alfazil and Anderson 2008; Garcia Boy et al. 2008; Henderson et al. 1997), and recently the United States Drug Testing Laboratory (USDTL) developed a liquid chromatography–mass spectrometry method for detection of PEth in DBS. Such analyses offer substantial advantages over existing methods, including (1) a minimally invasive method; (2) accuracy similar to samples collected by drawing blood; and (3) the low cost of sample collection, processing, transport, and storage (McDade et al. 2007). However, the validity of alcohol biomarkers in DBS specimens has yet to be established.

## Summary and Future Directions for Developing Novel Biomarkers of FASD

Despite a great deal of research to date, the goal of developing alcohol biomarkers that can detect lower levels of drinking and for longer intervals of time after the last drinking episode remain elusive. As illustrated in the figure, the vast majority of biomarkers that are sensitive enough to detect moderate and social drinking are present in body fluids/tissues for relatively short periods of time after the last drinking episode. This problem is compounded by the fact that, in general, many women who continue to drink during pregnancy will cut down on either the amount or frequency of drinking episodes, increasing the challenge of confirming a drinking history. In pregnant women, the use of biomarkers to identify risky drinking early in gestation presents a particular challenge, especially for those women who initiate prenatal care later in pregnancy. Thus, more sensitive biomarkers of alcohol metabolism, such as EtG/EtS, PEth, or FAEEs that can be detected in hair or other tissue types for a longer period after drinking could be very useful in clinical practice.

In addition, the systematic assessment of a profile of multiple biomarkers could increase the likelihood of detecting drinking during pregnancy and perhaps provide better insights about drinking patterns. It has been long recognized that a panel of biomarkers will detect alcohol exposure more accurately than a single biomarker (Bearer 2001; Stoler et al. 1998). However, none of the proposed panels have been widely used in clinical practice. Stoler and colleagues (1998) reported that among alcohol-abusing pregnant women, the risk of having an infant with growth retardation or facial features of FAS increased with an increasing number of positive test results with biological markers. To our knowledge, the validity of test batteries consisting of traditional biomarkers and direct alcohol metabolites has not been established in pregnant women.

A third area that warrants additional development is biomarkers for predicting fetal alcohol-induced damage. Very little attention has been directed towards the question of whether biomarkers of alcohol consumption or other biomarkers have utility as prognostic indicators for newborns at risk for adverse neurobehavioral outcomes. Among established alcohol biomarkers, a single study (Peterson et al. 2008) has correlated increasing meconium FAEE levels with poorer mental and psychomotor development in infants up to 2 years of age. Beyond established alcohol biomarkers, a single retrospective clinical study (Robinson et al. 1995) has examined the question of whether other chemical species might serve as biomarkers for FASD. These authors examined serum proteins in 12 children with FAS and 8 matched control children. Gel electrophoresis identified eight protein spots as candidate biomarkers for FAS. No single protein spot was significantly altered in all 12 FAS subjects, but the expression pattern of different combinations of four of the protein spots accurately identified all 12 FAS subjects. This study underscores the point that a signature pattern of changes in multiple chemical species may have greater diagnostic and prognostic capability than any single marker. Furthermore, high-throughput screening of proteins, or perhaps genes or other metabolites in biomarker tissue samples, affords a unique opportunity for developing more sensitive and specific biomarkers of FASD.

Finally, the development of more sensitive biomarkers of FASD based solely on clinical research continues to be extremely difficult largely as a result of the inherent variability in patient populations, the highly variable patterns of drinking among pregnant women, and the extended time intervals between an optimal time for collecting biological samples, such as during pregnancy or at birth, and the time at which measures of functional brain damage and/or adverse behavioral outcomes can be assessed. One alternative solution to this challenge is to first identify and validate biomarkers in established animal models of FASD and then, based on this information, pursue parallel human studies to assess clinical utility. Despite translational research challenges, the preclinical approach to novel biomarker development has four important advantages. First, preclinical studies increase the prospects of identifying novel markers without the confounding variables associated with patient populations. Second, biomarker validation can proceed in a more systematic and controlled fashion over a shorter period of time and in a more cost-effective manner. Third, an animal model system provides an opportunity to assess more directly how a biomarker signature may change as a function of alcohol dosing, patterns of exposure, and the influence of other interacting risk factors during pregnancy, such as nicotine, other drugs of abuse, stress, malnutrition, or heavy-metals exposure. How a biosignature pattern is altered by concurrent exposure to other risk factors would be critically important for the interpretation of data from clinical studies. Finally, an animal model system allows for direct correlation of biomarker patterns with markers of functional damage to the fetus and with longer-term adverse neurobehavioral outcomes. Such a model could, in the best-case scenario, provide insights about the mechanistic basis for the teratogenic damage, assessments that would be more difficult, if not impossible, to examine in a clinical study.

To date, two studies have used rodent models of FASD in combination with high-throughput screening procedures to initiate a search for novel biomarker candidates. Using a proteomic screening procedure, Datta and colleagues (2008) reported a significant increase in α-fetoprotein expression in amniotic fluid taken from gestational day (GD) 17 mouse fetuses after a single high-dose alcohol binge–like exposure on GD 8. Using a rat model of moderate drinking throughout pregnancy that produces offspring with learning deficits, Rosenberg and colleagues (2010) have identified 22 placental genes whose expression at term is significantly altered in alcohol-consuming dams. These alcohol-altered genes encode growth factors important for placental as well as fetal brain, vascular, endocrine, and immune system development. These findings illustrate the utility that these and other high-throughput screening procedures could have for developing more sensitive and specific biomarker systems both for the diagnosis of fetal alcohol exposure as well as for earlier prognostic indicators of fetal alcohol damage in exposed offspring.

## Figures and Tables

**Figure:**
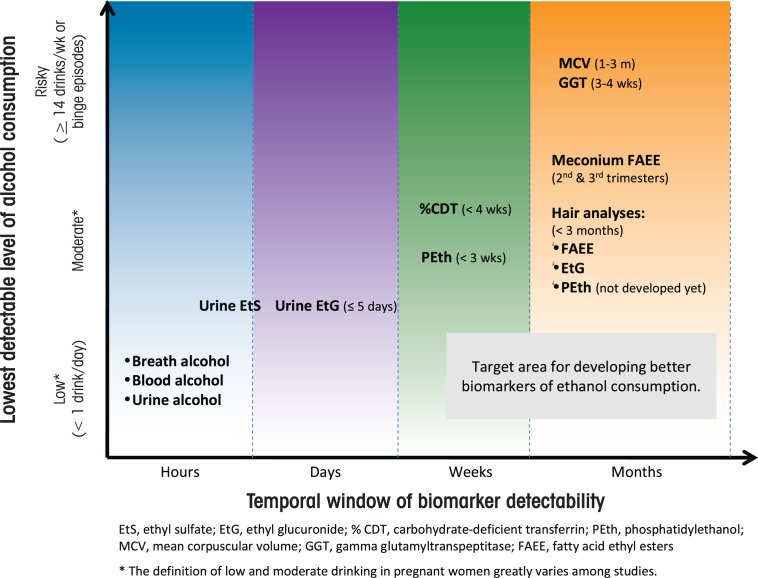
The period of time, or detection window, during which alcohol consumption can be detected and the lowest levels of alcohol consumption detectable by current alcohol biomarkers.

**Table:** Key Terms and Abbreviations

**FASD**	Fetal alcohol spectrum disorders
**FAS**	Fetal alcohol syndrome
**ARND**	Alcohol-related neurodevelopmental disorder
**Sensitivity**	The proportion of truly diseased persons in the screened population who are identified as diseased by the screening test
**Specificity**	The proportion of truly nondiseased persons who are so identified by the screening test
**TLFB**	Timeline follow-back procedure to assess alcohol exposure
**GGT**	γ-glutamyl transferase
**MCV**	Mean corpuscular volume
**CDT**	Carbohydrate-deficient transferrin
**FAEE**	Fatty acid ethyl esters
**EtG**	Ethyl glucuronide
**EtS**	Ethyl sulfate
**PEth**	Phosphatidylethanol

SOURCE: Adapted from Last, J.M. *A Dictionary of Epidemiology*. 4th ed. New York, NY: Oxford University Press, Inc., 2001

## Footnotes

1 TWEAK is a five-item scale developed to screen for risk drinking during pregnancy. It is an acronym for the following questions (Russell, 1994): T (tolerance)—“How many drinks can you hold?” W (worried)—“Have close friends or relatives worried or complained about your drinking in the past year?” E (eye-opener)—“Do you sometimes take a drink in the morning when you first get up?” A (amnesia)—“Has a friend or family member ever told you about things you said or did while you were drinking that you could not remember?” K/Cut Down—“Do you sometimes feel the need to cut down on your drinking?”

2 The CAGE questionnaire consists of four questions: 1. Have you ever felt you should cut down on your drinking? 2. Have people annoyed you by criticizing your drinking? 3. Have you ever felt bad or guilty about your drinking? 4. Eye opener: Have you ever had a drink first thing in the morning to steady your nerves or to get rid of a hangover?
